# The SIV Surface Spike Imaged by Electron Tomography: One Leg or Three?

**DOI:** 10.1371/journal.ppat.0020091

**Published:** 2006-08-25

**Authors:** Sriram Subramaniam

**Affiliations:** Scripps Research Institute, United States of America

Strategies to inhibit the cellular entry of human immunodeficiency virus type 1 (HIV-1) constitute an important element of current approaches to develop an effective vaccine against AIDS [1]. The entry of viruses such as HIV-1, and its closely related simian counterpart simian immunodeficiency virus (SIV), is mediated by the interaction of glycoprotein spikes on the viral membrane surface with receptors on the target cell membrane. The viral spike itself is a trimeric complex of gp41, a membrane protein which anchors the spike to the membrane, and gp120 which is non-covalently associated with gp41. Cartoon representations of the spike as a trimeric entity abound, but what does it really look like? So far, structural information on the spike has come largely from X-ray crystallographic analysis of the core of the gp120 monomer in the CD4-liganded [2] or unliganded forms [3], and of trimers of the helical regions of gp41 in a post-fusion conformation [4]. The structure of an intact trimeric spike itself has, nevertheless, remained elusive.

In this issue of *PLoS Pathogens,* Zanetti and coworkers [5] present electron tomographic studies of purified virions aimed at obtaining an average 3-D structure of the SIV surface spike. They report that the overall shape of the spike resembles that of a mushroom with a single stalk forming the junction with the viral membrane. By manually positioning the X-ray structure of an unliganded gp120 monomer [3] into this map, they suggest two atomic models for the probable structure of a trimeric spike displayed on the surface of their SIV specimens. This paper follows closely at the heels of a recent report by Zhu and coworkers [6], who have carried out almost exactly the same type of electron tomographic analysis of SIV, but conclude that the spike has a more globular “tripod-like” shape, with three separated legs near the surface of the viral membrane, and who propose a very different putative atomic model for the gp120 trimer. Nevertheless, both Zhu et al. and Zanetti et al. are confident that their models account for, and are consistent with, what is known about the structure of the spike from numerous biochemical experiments.

How is it possible that two different groups carrying out essentially the same experiment can arrive at completely different density maps for the viral spike, and contradictory models for the Env trimer? The simplest possibility is that there are genuine differences in the viruses imaged by the two groups. This, however, appears to be unlikely, given that both viruses are closely related and express very similar Env molecules that carry truncations in the C-terminal tail. There are subtle differences in the electron microscope hardware used by the two groups, but these differences are minor, and, indeed, the raw images obtained in both cases appear to be closely comparable. There are however, many aspects of data collection and image analysis that could have a profound impact on the final result. Here I attempt to provide a perspective on the possible origins of the discrepancy between the conclusions arrived at in these two papers, and focus primarily on experimental and computational aspects of using cryo-electron tomography that need careful attention to avoid artifacts and inaccuracies in structural determination.

Images recorded in a transmission electron microscope are “projection” images, meaning that they contain information from all regions of the specimen through which the beam was transmitted. Thus, each image contains information from all heights of the specimen collapsed into a single plane. From these projection images, one can derive 3-D information using one of two computational methods. In one method, often referred to as “single particle” electron microscopy [7], the poor signal-to-noise ratios inherent to biological electron microscopy are overcome by averaging images recorded from thousands of identical copies of specimens randomly oriented relative to the electron beam. The key to averaging multiple images is the requirement that the variations from one image to another are solely due to differences in its orientation relative to the electron beam. This type of averaging is even more powerful when the objects display intrinsic icosahedral, helical, or crystalline order. The second method, referred to as electron tomography [8,9], involves the imaging of “one-of-a-kind” objects such as nonsymmetric viruses and subcellular organelles. By recording a series of images in which the orientation of the specimen is varied relative to the incident beam, it is possible to obtain a series of projection views of the object (Figure 1), which can be converted into a 3-D volume using methods that generally rely on the use of weighted back-projection algorithms. Since the first use of these methods nearly four decades ago [10], they have been applied to a variety of biological objects to describe 3-D structures at varying resolutions. The two methods intersect in special cases as demonstrated in the work of Zhu et al. and Zanetti et al.: tomographic imaging of heterogeneous objects such as SIV can be combined with 3-D averaging of subvolumes of potentially identical entities such as the viral spike derived from multiple tomograms.

**Figure 1:**
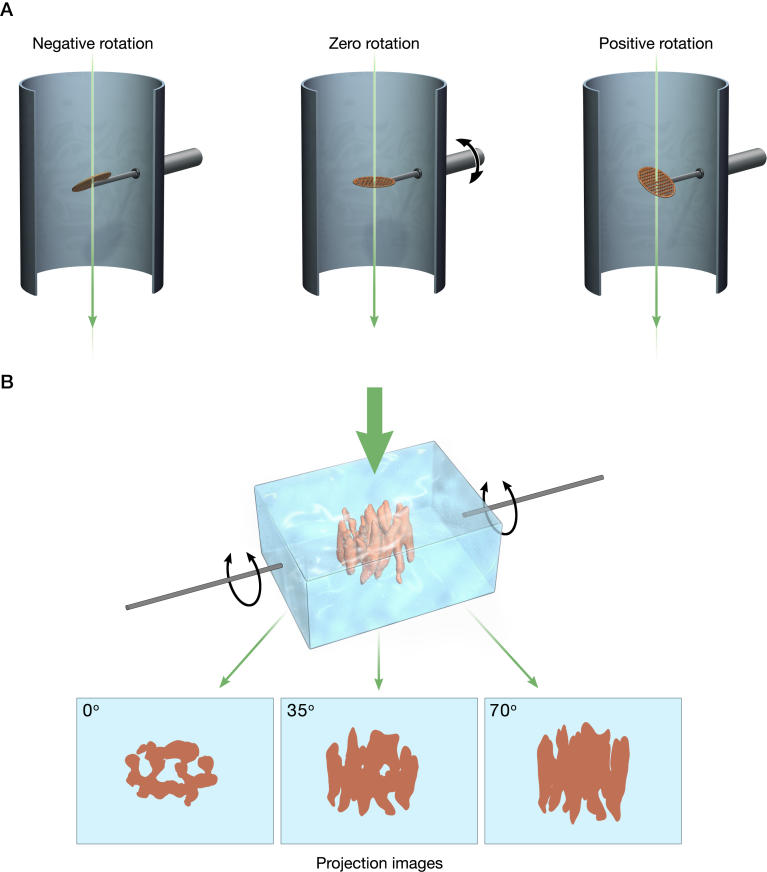
Principle of 3-D Imaging Using Electron Tomography (A) Schematic illustrating the principle of data collection for electron tomography by tilting specimens relative to the electron beam. (B) Rendering of three “perfect” projection views (at 0°, 35°, and 70°) generated by rotating a vitrified film of ice-embedded molecules relative to the electron beam. The structure shown is that of the oxalate transporter determined at 6.5 Å resolution using electron crystallography [18]. Structure determination by electron tomography involves starting with a set of projection images which are then effectively “smeared” out along their viewing directions to form back-projection profiles. These profiles are combined appropriately to recover the density distribution of the imaged object.

There are several important limitations to images obtained by current technologies for cryo-electron tomography that are particularly relevant to the results in these two papers. First, the physical limitations of the specimen stage and the microscope column usually restrict the maximal tilt that can be obtained to 70° for cryo-electron microscopic experiments, although higher tilts are possible in special cases. For tomograms obtained from single-axis tilts, there is therefore a missing “wedge” of data in reciprocal space and a corresponding loss in resolution in the vertical direction compared with the resolution obtained in the plane of the specimen. This scheme for data collection also results in anisotropic resolution within the plane, with the poorest resolution in a direction perpendicular to the tilt axis [11]. For example, in tomograms of a trimeric spike in which the long axis of the molecule at zero tilt is roughly along the direction of the electron beam, the resolution would be poorest along the length of the spike. Zanetti et al. account for this missing wedge in their analysis, while Zhu et al. do not.

Another constraint in cryo-electron tomography is that the electron doses needed to obtain tomograms are higher than those normally used in cryo-electron microscopy of single particles or 2-D crystals where the highest resolutions have been obtained. In structures determined by averaging using single particle electron microscopy, as used for example in structural analysis of the ribosome [12], specimens are typically imaged with a single exposure at electron doses of 10–20 electrons/Å^2^. The electron doses used by both groups reporting analysis of SIV are many times higher, but this could be considered a reasonable compromise given present-day technology, because lower doses may not produce tomograms with the sufficiently high signal-to-noise ratios. However, the consequence of the higher radiation damage resulting from these higher doses in electron cryo-electron tomography is not very well understood, and could contribute to image heterogeneity. Improved contrast can also be generated by recording images at higher defocus values. This approach has its own problems, since methods for correction of the contrast transfer function of the electron microscope, which are very well established in structural analysis using single particle microscopy, are not yet in common use for electron tomography. Proper correction of this contrast transfer function will be essential for reliable molecular interpretation of cryo-electron tomographic images.

Yet another challenge concerns the choice of computational strategies for averaging in the context of tomography to obtain higher signal-to-noise ratios, as in the case of viral spike analysis. To average several noisy images, one needs a reference image. The choice of this reference is extremely important, because this can irreversibly bias all subsequent steps in analysis. This is further complicated by the presence of the missing wedge, which is in different orientations for each subvolume that is being averaged. This can lead to further errors in alignment in addition to those resulting from intrinsic noise in the images. Zanetti et al. report that they obtain the same final map starting from a different starting model for alignment, and this type of control is useful for exploring obvious effects of model bias. However, these are early days in this field, and many more controls will need to be done to establish the reliability of structures derived by averaging electron tomographic data. Inevitably, the way forward will involve rigorous and exhaustive classification of large image datasets to establish which classes of images are close enough to be averaged. This is likely to be critical not only for understanding viral spike architecture, but to address fundamental questions such as the extent of variation between different subsets of spikes on the viral membrane and between different types of viruses.

The presence of internal symmetry which can be used to improve signal-to-noise ratios can also be a double-edged sword: it helps when there is certainty that the symmetry is real but will lead to artifacts if symmetrization is applied to average dissimilar entities [13]. In the case of the viral spike, both Zhu et al. and Zanetti et al. enforce strict 3-fold symmetry in the spike from the very early stages of refinement. While there is overwhelming evidence that the viral spike is trimeric, the extent of variation, if any, between individual monomers is far less certain. In fact, there already exists biochemical evidence for conformational variation in monomeric and trimeric HIV-1 envelope glycoproteins [14]. In the absence of independent validation for the use of symmetry, substantial errors could be introduced by aligning all of the data to a single symmetrized reference.

Finally, there is always the question of how good a map needs to be before it can be reliably interpreted in terms of the atomic structures of its subcomponents. Positional accuracy is a resolution-dependent quantity. In single particle electron microscopy, there has been great success in interpreting maps at resolutions below 10 Å in terms of the locations of tertiary and secondary structural elements [15,16]. Similarly, maps obtained using electron crystallography can also be interpreted in terms of secondary structural elements when the resolution is sufficiently high, as in the case of the bacterial oxalate transporter (Figure 1B) [17].

In biological electron tomography, this is largely uncharted territory, even assuming that the missing wedge has been taken into account. In the case of the viral spike, it is further complicated by the fact that 50% of the mass comes from carbohydrate moieties, and there is no way at present to assess what contribution they make to the overall density map as compared with the polypeptide components of the spike. The other challenge is that only two-thirds of the polypeptide portion, and about 10% of the carbohydrates, are represented in the currently available model at atomic resolution of the gp120 [3]. Given these limitations, there will undoubtedly be significant errors in positioning the known components of gp120 structure into density maps of the spike at the present resolution. Because of these considerations, the accuracy of the fits presented by both Zanetti et al. and Zhu et al. is likely to be limited.

So, in summary, is the viral spike shaped like a tripod with three legs as Zhu et al. have it, and is thought to be the case for murine leukemia virus [17]? Or is it shaped like a mushroom with one stalk, as Zanetti et al. have it? Or something in between? Is the V3 loop buried at the trimer interface or does it face outward? Is the co-receptor binding site occluded because it is buried at the trimer interface, or do other regions of gp120 generate steric hindrance? What is the distribution of carbohydrates on the surface? These questions are at the heart of rational vaccine design efforts, but answers to these questions will require data at higher resolution and the use of 3-D image classification strategies that can distinguish differences due to noise from those due to conformational variation. It will also be essential to obtain density maps of Env spike complexes in the presence of antibodies to various epitopes on the spike. Despite all of these caveats, Zhu et al. and Zanetti et al. should be lauded for their efforts in pioneering the use of electron tomography to tackle the complex problem of SIV and HIV spike architecture. Rapid advances are being made in hardware and software for cryo-electron tomography, and there is much hope that 3-D images obtained using this approach will not need to be confined to resolutions that are too modest for reliable interpretation in terms of atomic structures of individual components. It is safe to say that we have barely scratched the surface, and significant progress may be expected in the near future.

## Abbreviations

SIV: simian immunodeficiency virus

## References

[ppat-0020091-b001] Douek DC, Kwong PD, Nabel GJ (2006). The rational design of an AIDS vaccine. Cell.

[ppat-0020091-b002] Kwong PD, Wyatt R, Majeed S, Robinson J, Sweet RW (2000). Structures of HIV-1 gp120 envelope glycoproteins from laboratory-adapted and primary isolates. Structure.

[ppat-0020091-b003] Chen B, Vogan EM, Gong H, Skehel JJ, Wiley DC (2005). Structure of an unliganded simian immunodeficiency virus gp120 core. Nature.

[ppat-0020091-b004] Caffrey M, Cai M, Kaufman J, Stahl SJ, Wingfield PT (1998). Three-dimensional solution structure of the 44 kDa ectodomain of SIV gp41. EMBO J.

[ppat-0020091-b005] Zanetti G, Briggs JAG, Grunewald K, Sattentau Q, Fuller SD (2006). Cryo-electron tomographic structure of an Immunodeficiency Virus Envelope complex in situ test. PLoS Pathog.

[ppat-0020091-b006] Zhu P, Liu J, Bess J, Chertova E, Lifson JD (2006). Distribution and three-dimensional structure of AIDS virus envelope spikes. Nature.

[ppat-0020091-b007] van Heel M, Gowen B, Matadeen R, Orlova EV, Finn R (2000). Single-particle electron cryo-microscopy: Towards atomic resolution. Q Rev Biophys.

[ppat-0020091-b008] Baumeister W (2002). Electron tomography: Towards visualizing the molecular organization of the cytoplasm. Curr Opin Struct Biol.

[ppat-0020091-b009] Subramaniam S (2005). Bridging the imaging gap: Visualizing subcellular architecture with electron tomography. Curr Opin Microbiol.

[ppat-0020091-b010] DeRosier DJ, Klug A (1968). Reconstruction of three-dimensional structures from electron micrographs. Nature.

[ppat-0020091-b011] Mastronarde DN (1997). Dual-axis tomography: An approach with alignment methods that preserve resolution. J Struct Biol.

[ppat-0020091-b012] Mitra K, Frank J (2006). Ribosome dynamics: Insights from atomic structure modeling into cryo-electron microscopy maps. Annu Rev Biophys Biomol Struct.

[ppat-0020091-b013] Jiang W, Chang J, Jakana J, Weigele P, King J (2006). Structure of epsilon15 bacteriophage reveals genome organization and DNA packaging/injection apparatus. Nature.

[ppat-0020091-b014] Yuan W, Bazick J, Sodroski J (2006). Characterization of the multiple conformational states of free monomeric and trimeric human immunodeficiency virus envelope glycoproteins after fixation by cross-linker. J Virol.

[ppat-0020091-b015] Borgnia MJ, Shi D, Zhang P, Milne JL (2004). Visualization of alpha-helical features in a density map constructed using 9 molecular images of the 1.8 MDa icosahedral core of pyruvate dehydrogenase. J Struct Biol.

[ppat-0020091-b016] Ludtke SJ, Chen DH, Song JL, Chuang DT, Chiu W (2004). Seeing GroEL at 6 A resolution by single particle electron cryomicroscopy. Structure.

[ppat-0020091-b017] Hirai T, Heymann J, Shi D, Sarker R, Maloney PC (2002). Three dimensional structure of a bacterial oxalate transporter. Nat Struct Biol.

[ppat-0020091-b018] Forster F, Medalia O, Zauberman N, Baumeister W, Fass D (2005). Retrovirus envelope protein complex structure in situ studied by cryo-electron tomography. Proc Natl Acad Sci U S A.

